# LB8. Lower SARS-CoV-2 IgG and Pseudovirus Neutralization Titers Post-mRNA Vaccination among People Living with HIV

**DOI:** 10.1093/ofid/ofab466.1639

**Published:** 2021-12-04

**Authors:** Matthew A Spinelli, Michael J Peluso, Kara Lynch, Cassandra Yun, David V Glidden, Timothy J Henrich, Steve Deeks, Monica Gandhi

**Affiliations:** 1 University of California, San Francisco, San Francisco, CA; 2 University of California San Francisco, San Francisco, California; 3 UCSF, San Francisco, California

## Abstract

**Background:**

Limited data are available on whether there are differences in the immune response to SARS-CoV-2 vaccination by HIV status or by mRNA vaccine type.

**Methods:**

We saved residual outpatient laboratory samples of all previously mRNA-vaccinated individuals in the adult medicine clinics of a public hospital with a large outpatient HIV clinic during May 2021, and then excluded individuals with prior SARS-CoV-2 infection. We next 1:1 matched 100 PLWH to 100 outpatient HIV-negative adult medicine patients receiving care for chronic medical conditions on days since completion of second vaccination (minimum 10), sex, age +/-5 years, and the type of mRNA vaccine received. We defined a non-response as reciprocal pseudovirus neutralizing titer< 10 and anti-RBD IgG< 10 relative fluorescent units, and compared non-response by HIV status using mixed models.

**Results:**

In each matched group there were 13 women; 25 received the mRNA-1273 vaccine and 75 received the BNT162b2 vaccine; the median age was 59. The median time from second vaccination was 35 days (IQR: 20–63). Among PLWH, the median CD4+ T-cell count was 511 (IQR: 351–796) and 5 individuals had HIV RNA > 200.

We found 2.4-fold greater odds of pseudovirus neutralizing antibody non-response among PLWH compared to people without HIV (95% CI=1.1–5.4). Although few individuals in each group did not mount an IgG response (12 among PLWH vs. 5; p=0.08), continuous anti-RBD IgG concentrations were 43% lower among PLWH (95% CI=0.36–0.88).

Among PLWH, when adjusting for age, sex, and days post-vaccination, each 100-cell increase in CD4+T-cell count was associated with 22% higher neutralizing antibody titers (GMR 1.22; 95% CI=1.09–1.37). Unsuppressed HIV RNA >200 was associated with 89% lower neutralizing antibody titers (GMR 0.11; 95% CI=0.01–0.84). Receipt of the BNT162b2 vs. mRNA-1273 vaccine was associated with 77% lower neutralizing titers (GMR 0.23; 95% CI=0.08–0.65) among PLWH.

Post-mRNA Vaccination SARS-CoV-2 IgG Concentrations and Pseudovirus Neutralizing Titers by HIV Status and Vaccine

**Conclusion:**

PLWH had lower than expected response to mRNA SARS-CoV-2 vaccines, with the highest non-response among those with low CD4+ counts, unsuppressed HIV RNA, and those who received the BNT162b2 vaccine. Immunization strategies to improve immune responses among PLWH should be studied, and may include booster vaccination or preference of the mRNA-1273 vaccine in this group.

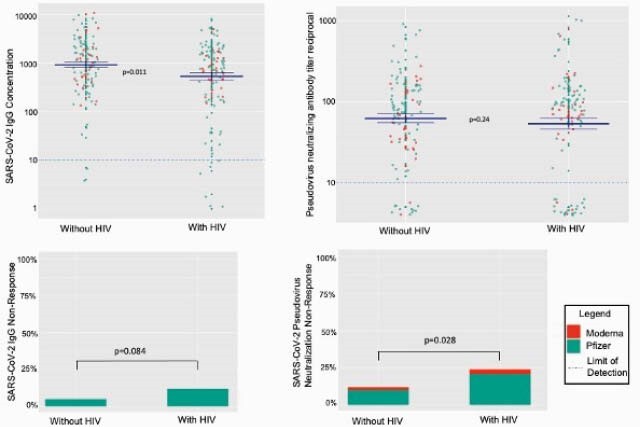

**Disclosures:**

**Matthew A. Spinelli, MD, MAS**, Nothing to disclose **Monica Gandhi, MD, MPH**, Nothing to disclose

